# Adenosine Depletion as A New Strategy to Decrease Glioblastoma Stem-Like Cells Aggressiveness

**DOI:** 10.3390/cells8111353

**Published:** 2019-10-30

**Authors:** Ignacio Niechi, Atenea Uribe-Ojeda, José Ignacio Erices, Ángelo Torres, Daniel Uribe, José Dellis Rocha, Pamela Silva, Hans G. Richter, Rody San Martín, Claudia Quezada

**Affiliations:** 1 Instituto de Bioquímica y Microbiología, Facultad de Ciencias, Universidad Austral de Chile, Valdivia 511-0566, Chile; ignacio.niechi@gmail.com (I.N.); atenea.uribe@gmail.com (A.U.-O.); ignacioern@gmail.com (J.I.E.); astalpm@gmail.com (Á.T.); Daleuri@hotmail.com (D.U.); jdellis.rocha@gmail.com (J.D.R.); pamesilvalv@gmail.com (P.S.); 2 Instituto de Anatomía, Histología y Patología, Facultad de Medicina, Universidad Austral de Chile, Valdivia 511-0566, Chile; hrichter@uach.cl

**Keywords:** glioblastoma, adenosine, invasiveness, stemness, adenosine deaminase

## Abstract

Glioblastoma is the brain tumor with the worst prognosis. This is mainly due to a cell subpopulation with an extremely aggressive potential, called glioblastoma stem-like cells (GSCs). These cells produce high levels of extracellular adenosine, which are increased even more under hypoxic conditions. Under hypoxia, adenosine signaling is related to HIF-2α expression, enhancing cell aggressiveness. Adenosine can be degraded using recombinant adenosine deaminase (ADA) to revert its pathological effects. The aim of this study was to degrade adenosine using ADA in order to decrease malignancy of GSCs. Adenosine depletion was performed using recombinant ADA. Migration and invasion were measured by transwell and matrigel-coated transwell assay, respectively. HIF-2α-dependent cell migration/invasion decreased in GSCs treated with ADA under hypoxia. MRPs-mediated chemoresistance and colony formation decreased in treatment with ADA. In conclusion, adenosine depletion using adenosine deaminase decreases GSCs aggressiveness.

## 1. Introduction

Glioblastoma (GBM) is the most common and aggressive brain tumor with enhanced proliferation, chemoresistance and invasive potential [1,2,3]. Despite the multimodal treatments including tumor resection followed by radiotherapy and chemotherapy [2,4,5], there has been no improvement in the survival rates, due to the early and inevitable tumor recurrence [6,7,8]. Recurrence is mediated mainly by a cellular subpopulation with stem cells characteristics called glioblastoma stem-like cells (GSCs) [9]. These cells can be found in specific niches within the tumor, associated with blood vessels with an appropriate oxygen supply or under hypoxic regions associated with necrosis [10]. Perivascular GSCs maintain its stem phenotype mainly through Notch and Hedgehog pathways from endothelial cells, promoting angiogenesis and tumor growth through VEGF secretion [10]. On the other hand, hypoxic GSCs have been related to a highly aggressive phenotype due to its ability to mediate resistance to chemotherapeutic drugs [7] and to infiltrate healthy tissue [11]. Tumor infiltration is mediated by the expression of epithelial-mesenchymal transition markers, cell invasion/migration and expression of matrix metalloproteases (MMPs) [11,12,13]. GSCs invasiveness is due in part to the biological activity of adenosine, a nucleoside up-regulated in GBM tumor microenvironment which production is aberrantly increased in GSCs [10,14,15]. The extracellular adenosine production pathway is mediated by ATP or ADP dephosphorylation to AMP by CD39 and the subsequent hydrolysis to adenosine by CD73 and/or PAP ectonucleotidases [12]. Adenosine is irreversibly degraded to inosine by the action of adenosine deaminase (ADA), reverting its patho-physiological effects [16]. ADA is indeed used for the treatment of SCID, a disease characterized by high levels of systemic adenosine, reverting its pathological signaling [16,17,18]. Adenosine-producing enzymes have been found to be also increased in differentiated GBM cell lines, primary cultures and GSCs [9,19,20]. High levels of CD73 are correlated with the enhanced migratory and invasive capacity of glioma cells [11,14,21] and also its inhibition decreases cellular invasion in breast cancer cells [22]. Recently it was demonstrated that PAP is involved in adenosine-dependent EMT markers expression, migration and GSCs invasion [11,23]. Four adenosine receptors (ARs) have been described; two of high affinity activated mainly at nM adenosine concentrations (A_1_ and A_2A_) and two of low affinity activated at μM adenosine concentrations (A_2B_ and A_3_) [24]. The physiological concentration of extracellular adenosine in several tissues is 30-200 nM [24,25], activating A_1_ and A_2A_; however, within tumor microenvironment adenosine concentration increases up to 100 times, triggering its effects through its low affinity adenosine receptors A_2B_ and A_3_ [10,19,26,27]. Compared to healthy tissue, increased expression of these receptors has been observed in breast, rectum and GBM, among other tumors, so they have been targeted in preclinical studies demonstrating that the use of agonists may promotes the tumor progression of several cancer types, including melanoma, prostate, colon and hepatocellular carcinoma [10,11,15,19]. In GBM there is increased but not homogeneous vascularization which limits the availability of oxygen generating hypoxic niches where GSCs persist [28,29]. The hypoxic condition controls the expression of several genes, including Hypoxia-Inducible Factors (HIFs) [30,31]. Hypoxia and HIFs have been related to the invasive capacity of GBM cells and GSCs [26,29,32,33,34], furthermore, the stabilization of HIF-2α promotes the expression of PAP-dependent adenosine production of GSCs under hypoxia [26]. The transcriptional activity of HIF-1α is mainly related to metabolic pathways [35], while HIF-2α has been mainly related to the expression of proteins involved in cell migration/invasion processes, such as MMPs [34,36,37]. Although both HIFs are important in the maintenance of the stem phenotype in different models, there is consensus that HIF-2α is more specific and selective for GSCs [33]. Interestingly, HIF-2α, and not HIF-1α, would be involved in the migration and invasion of GSCs under hypoxic conditions [11]. GBM recurrence is also mediated by its enhanced chemoresistant phenotype, especially in GSCs [38,39]. Chemoresistance is mainly due to the multiple-drug resistance (MDR) phenomenon that includes overexpression of several ABC-transporters such as multidrug resistance-associated proteins (MRPs), which extrude drugs to the extracellular medium, thereby enhancing chemoresistance to drugs, such as vincristine [9,40]. The expression and activity of MRPs is positively regulated by adenosine through its A_3_AR receptor in GSCs, suggesting that its effects could be regulated by adenosine depletion [9]. Here, we evaluated the effect of the enzyme that degrades adenosine (ADA) on adenosine-dependent invasive capacity of GSCs mediated by HIF-2α under hypoxia conditions and MRPs-mediated chemoresistance, as a new strategy proposed to revert GBM recurrence.

## 2. Materials and Methods

### 2.1. Cell Culture

U87MG cells were grown in neurobasal medium (Gibco, Waltham, MA, USA) supplemented with EGF (20 ng/mL; Peprotech^©^, Rocky Hill, NJ, USA), bFGF (20 ng/mL; Peprotech^©^), 1X B27 (Gibco ™), 1× Glutamax (Gibco) and penicillin/streptomycin (100 U/mL, Gibco) at 37 °C. After 5 days of culture GSCs were plated to carry out different tests and treatment. GSCs primary culture were obtained from surgical human GBM samples and cultured with M21 medium DMEM/F12 (Gibco); 1X MEM non- essential amino acids (Gibco™); Hepes (1 M; Gibco); D-Glucose (45%; Sigma, Darmstadt, Germany); BSA Fraction V (7.5%; ThermoFisher); Sodium pyruvate (100 mM; ThermoFisher); L-glutamine (200 mM; Gibco); penicillin/streptomycin (100 U/mL, Gibco); hydrocortisone (0.6 µg/mL; Sigma); triiodothyronine (0.1 mg/mL; Sigma) and 1X N1 supplement (Sigma) supplemented with EGF (25 ng/μL; Sigma), bFGF (25 ng/μL; Sigma) and heparin (0.5 μg/mL; Sigma). normoxia (21% O_2_) and hypoxia (0.5% O_2_) conditions were generated using a gas chamber (5% CO_2_ and 95% N_2_ mixture) during 24 h.

### 2.2. Adenosine Quantification

GSCs were cultured and treated with ADA 1U/mL by 24 h. Culture medium was collected and deproteinization was performed with TCA and then filtered. Medium without proteins was treated with 2-chloroacetaldehyde to induce nucleosidederivatization and HPLC fractionation was performed in a Chromolith Performance RP-18 column (Merck, Darmstadt, Germany) coupled to a fluorescence spectrometer as described elsewhere [20].

### 2.3. Western Blot

Proteins (40 µg) were separated by SDS-PAGE (BioRad, Hercules, CA, USA), transferred to 0.45 µm nitrocellulose membranes and blocked with 1 × PBS/0.05% tween20/BSA 5% for 1 h. Membranes were incubated with primary antibodies overnight at 4 °C, followed by secondary antibodies HRP-conjugate during 1 h. Anti-Snail (Cell Signaling, 1:1,000, Danvers, MA, USA), anti-MMP-9 (Santa Cruz, 1:1,000, Dallas, TX, USA), anti-β-actin (Cell signaling, 1:5,000), anti-HIF-2α (Cell signaling, 1:1,000), anti-Zeb1 (Cell Signaling, 1:1,000), anti Twist1 (Cell Signaling, 1:1,000) anti E-cadherin (Cell Signaling, 1:1,000). HRP-conjugated secondary antibodies were purchased from Jackson Laboratories and used at 1:50,000 in 1X PBS-T 0.05%. Bands were visualized by West Dura chemiluminescence system (ThermoFisher) and analyzed in a Syngene G: Box equipment (Synoptics, Cambridge, UK).

### 2.4. RT-qPCR

Total RNA was extracted with TRIzol (Gibco) and quantified by NanoDrop. Reverse transcription was performed with 1 µg RNA plus MMLV-RT (ThermoFisher) following manufacturer instructions. qPCR was performed using the ΔΔCt method and ACTB (β-actin) as a normalizer gene using buffer 2x Master mix qPCR Brilliant II Sybr ^®^ Green (ThermoFisher, Waltham, MA, USA), following the manufacturer’s instructions. Primers used were: EPAS1 (HIF-2α) F:5′-gacaaggtctgcaaagggttttgg-3′ R:5′-ggaaggcttgctcttcatactcca-3′; MMP9 F: 5′-atttctgccaggaccgcttctact-3′ R: 5′-tgtcataggtcacgtagcccactt-3′; TWIST1 F: 5′-tcagccactgaaaggaaaggca-3′ R: 5′-gcaggccagtttgatc ccagtatt-3′; SNAIL F: 5′-cttctcactgccatggaattccct-3′ R: 5′-tccacagaaatggccatgggaa-3′

### 2.5. Protein Stability

GSCs (1 × 10^6^) were seeded into 12 well plates and cultured overnight at 37 °C under hypoxia with 20 μg/mL cycloheximide (CHX) in the absence or presence of 1 U/mL ADA between 0–30 min or 0–4 h. Cells were harvested after treatment, lysed and 40 μg of total protein were analyzed by western blot.

### 2.6. Cell Adhesion

Cells were seeded in a 96-well plate (2.5 × 10^5^ cells/well) pre-treated with 2 µg/mL fibronectin incubated 1 h at 37 °C and incubated under hypoxia. Cells were treated with 1 U/mL ADA for 0–30 min. Cells were stained for 15 min with 1% crystal violet/20% methanol and washed twice with 1X PBS. Cell adhesion was analyzed by measuring the optical density at 600 nm in a microplate reader (Synergy, BioTek, Winooski, VT, USA).

### 2.7. Cell Migration and Invasion

GSCs were plated (75,000 cells/chamber) on the top side of a polycarbonate Transwell chamber (Corning, Lowell, MA, USA) for migration assay or in a 300 µg/mL matrigel-coated Transwell chamber (Corning) for invasion assay. Cells were seeded in serum-free neurobasal medium for U87 or M21 for primary cultures. As chemoattractant medium was used 10% FBS DMEM-F12 in the bottom chamber. Cells were incubated at 37 °C for either 6 h or 12 h for migration or invasion assays, respectively. Cells were stained for 15 min with 1% crystal violet/20% methanol, after that were washed with water and in the top chamber were carefully removed with cotton swabs. Cells were counted using 10x objective in 5 different fields of the underside of the insert. Cells mean number was normalized to 1 using the control conditions and then plotted.

### 2.8. Zymography Assay

U87-GSCs medium was collected after hypoxia and ADA treatments and centrifuged at 12,000× *g* for 20 min. Pellet was discarded and proteins were loaded in a 7.5% polyacrylamide gel plus 10 mg/mL of gelatin. Gel was washed by 1 h with 2.5% triton X100, 50 mM Tris-HCl, 5 mM CaCl_2_ and 50 µM ZnCl_2_ by 20 min 2 times. Then was incubated with 50 mM Tris-HCl, 5 mM CaCl_2_ and 50 µM ZnCl_2_ overnight at 37 °C. Gel was dyed with 0.25% Coomassie Blue R250, 10% acetic acid, 40% methanol by 1 h at room temperature and washed with acetic acid and methanol until the appearance of bands, changing the solution every 10 min.

### 2.9. MRPs Activity

MRPs activity was evaluated as described elsewhere [9]. GSCs (2 × 10^5^) were seeded in DMEM/F-12 serum free for 24 h at 37 °C in 24-well plates and treated with 1 U/mL ADA. Cells were incubated with 500 nM of CFDA for 15 min and washed three times with 1X PBS and incubated for 15 min in serum-free DMEM/F-12 medium to promote CFDA extrusion. Cells were washed three times with 1X PBS and fluorescence of cell extracts was measured by flow cytometry (FACS Jazz; BD Biosciences, Franklin Lakes, NJ, USA).

### 2.10. Cell Viability Assay

CellTiter 96^®^ AQueous One Solution Cell Proliferation Assay (MTS) from Promega (Madison, WI, USA) was performed following manufacturer instructions. Briefly, GSCs (10 × 10^4^) were seeded in 96-well plates for 24 h and treated with Vincristine (100 nM) alone or in combination with 1U/mL ADA for 24 h. Cells were incubated with MTS reagent for 2 h and absorbance was measured at 550 nm using a microplate reader (Synergy HT, BioTek Instruments, Inc.).

### 2.11. Soft Agar Colony Formation Assay

Bottom layer of semi-solid agar composed of 0.75 mL of 1% soft agar and 0.75 mL of 2X DMEM was added per well of a 6-well plate. A mixture of 0.75 mL of 0.6% agar and 0.75 mL of M21 2X containing 5,000 PC-GSCs per well was added on the bottom layer. Cells were refreshed every 3 days with culture medium for 21 days and stained with a mixture of 0.05% violet crystal/25% methanol. Colonies were counted and plotted.

### 2.12. Statistical Analysis

Plotting and statistical analysis were performed in GraphPad Prism software. Data were plotted as mean ± SD from at least three independent experiments. Statistical analysis was performed with Peritz F multiple means comparison test. Student’s t-test was used for unpaired data. * *p* < 0.05 means statistically significant.

## 3. Results

### 3.1. Adenosine Depletion Decreases HIF-2α Levels Under Hypoxia

#### 3.1.1. Adenosine Deaminase Decrease Extracellular Adenosine Levels

**Figure 1:**
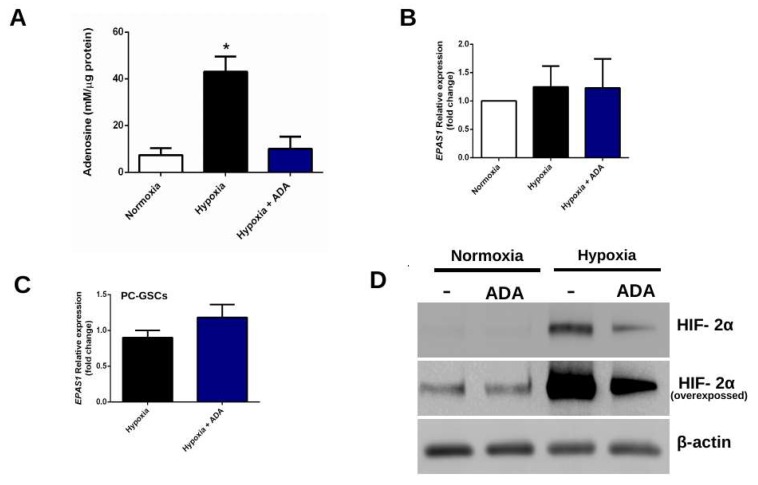
HIF-2α levels under hypoxia and adenosine depletion. (**A**) Extracellular adenosine quantification of U87-GSCs under hypoxia treated with 1 U/mL ADA for 24 h. Adenosine concentration (nM) was normalized to total protein levels (μg) (**B**) *EPAS1* (HIF-2α) transcript levels of U87-GSCs under hypoxia and 1 U/mL ADA for 24 h were measured by RT-qPCR (**C**) Same as in B, but with PC-GSCs (**D**) HIF-2α protein levels of U87-GSCs under normoxia or hypoxia treated with 1 U/mL ADA for 24 h were analyzed by western blot. *n* = 3, *p < 0.05.

Glioblastoma stem-like cells (GSCs) produce high levels of adenosine and its extracellular concentrations increase even further under hypoxia conditions (Figure 1A). In order to degrade adenosine, GSCs derived from U87MG cell line (U87-GSCs) were incubated with recombinant adenosine deaminase (ADA) for 24 h. Adenosine levels were quantified by HPLC fractionation coupled to a fluorescence spectrophotometer and it was established that 1 U/mL of ADA was sufficient to decrease extracellular levels of adenosine by 75% (Figure 1A).

#### 3.1.2. Adenosine Deaminase Decreases HIF-2α Protein But Not mRNA Levels

Since HIF-2α levels are stabilized under hypoxia and it has a role in adenosine-dependent cell invasion, we evaluated the effect of adenosine depletion with 1 U/mL ADA on HIF-2α levels in GSCs. HIF-2α transcript levels (*EPAS1*) displayed a tendency towards an increase under hypoxia in U87-GSCs (Figure 1B), however adenosine depletion with ADA was not able to revert this pattern in U87-GSCs (Figure 1B) nor in GSCs derived from primary culture (PC-GSCs) (Figure 1C). As expected, HIF-2α protein levels were increased in hypoxic U87-GSCs and surprisingly this was prevented by adenosine depletion (Figure 1D). Together, these results suggest an adenosine-mediated post-transcriptional regulation of HIF-2α under hypoxia.

### 3.2. Adenosine Depletion Inhibits HIF-2α Stability Under Hypoxia

**Figure 2:**
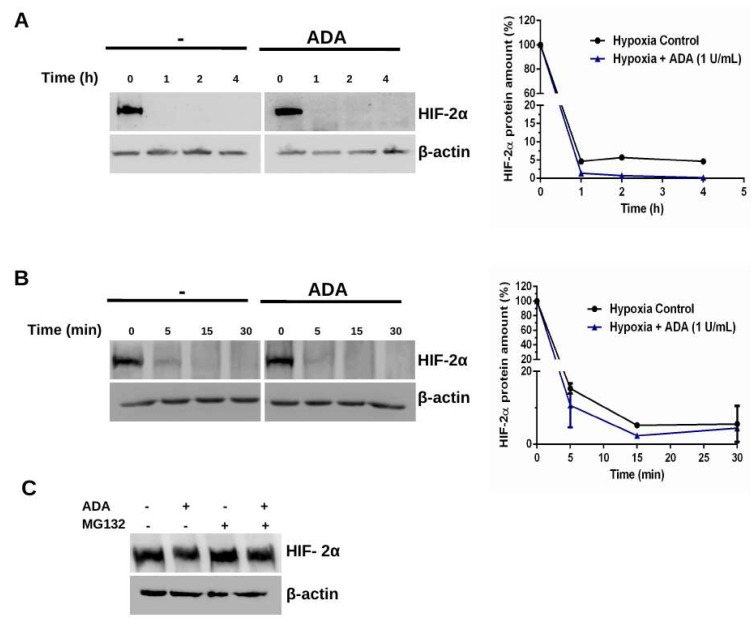
HIF-2α protein stability. (**A**) Hypoxic U87-GSCs were treated with 20 μg/mL CHX in absence or presence of 1 U/mL ADA for 0–4 h. Protein levels were analyzed by western blot and then plotted. (**B**) Same as in **A**, but ADA treatment was between 0–30 min. (**C**) Hypoxic U87-GSCs were treated with 10 μM MG132 and/or 1 U/mL ADA for 24 h and proteins were analyzed by western blot. *n* = 3.

Previous results suggest that HIF-2α regulation by adenosine is not related to transcriptional activity; thus, a protein stability assay was performed with cycloheximide (CHX). It was not possible to distinguish significant time-dependent variations on protein stability, probably due to the fast HIF-2α degradation between 0-4 h (Figure 2A). When trying shorter times, 0–30 min, protein levels decreased markedly at 5 min of treatment with CHX in U87-GSCs and disappeared almost completely at 15 min (Figure 2B). Apparently, this effect is faster with ADA, but no significant differences were observed (Figure 2B). Due to high degradation rate, a protein accumulation assay was performed in presence of the proteasome inhibitor MG-132 in U87-GSCs. As expected, HIF-2α protein levels were accumulated by inhibiting proteasome degradation, however this was not reversed with ADA. Given that ADA decreased protein but not HIF-2α mRNA levels, while it was not able to reverse its accumulation under MG-132 treatment, these results strongly suggest that adenosine may regulate HIF-2α proteasomal degradation (Figure 2C).

### 3.3. Adenosine Depletion Decreases GSCs Hypoxia-Dependent Cell Adhesion and Migration

It is well established that hypoxia is a microenvironmental factor that enhances GSCs adhesion and migration due to several factors, such as aberrant extracellular adenosine accumulation and HIF-2α stabilization. To counteract this, a fibronectin-dependent cell adhesion assay was performed between 0 and 30 min with hypoxic U87-GSCs with ADA treatment. 

**Figure 3:**
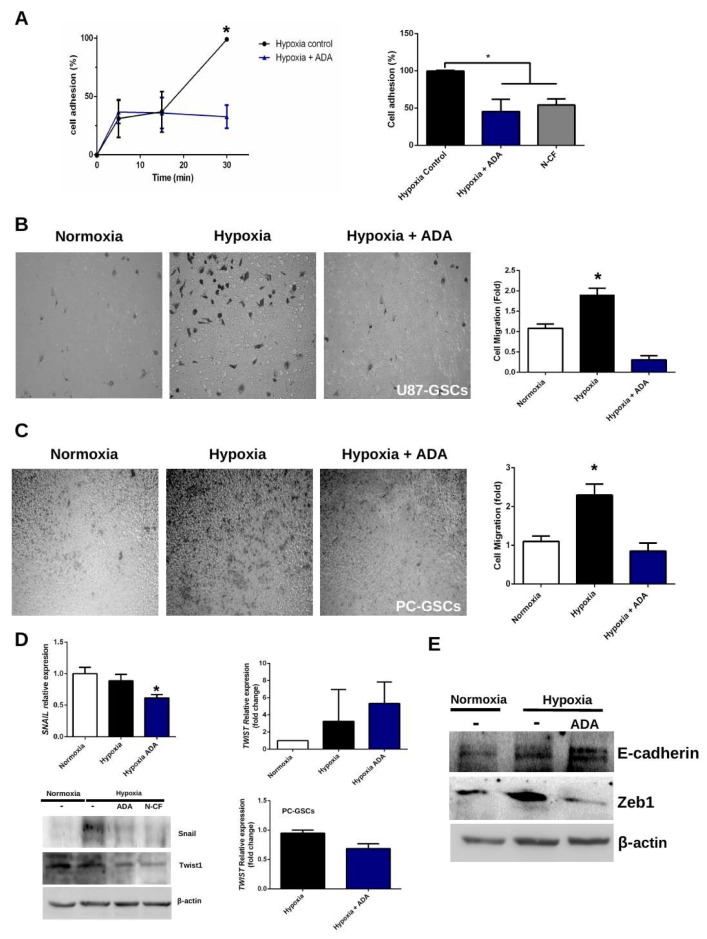
Effects of adenosine depletion on GSCs adhesion and migration. (**A**) U87-GCSs were seeded and immediately treated with 1 U/mL ADA for 0-30 min. Cell adhesion was plotted as percentage (left). Cell adhesion was measured at 30 min plus HIF-2α antagonist 10 μM N-CF (right). (**B**) 3D-transwell migration assay of U87-GCSs in absence or presence of 1 U/mL ADA for 6 h. (**C**) Same as in **B**, but using PC-GSCs. Cell migration was plotted as fold change of the normoxic condition. (**D**) Snail and Twist1 levels were measured in hypoxic GSCs treated with 1U/mL ADA by RT-qPCR and western blot plus HIF-2α antagonist 10 μM N-CF. (**E**) EMT markers (E-cadherin and Zeb1) were evaluated by western blot in U87-GSCs. *n* = 3 * *p* < 0.05.

Here we show that at 30 min of ADA treatment, only ~25% of the cells were adhered to the fibronectin-coated plate compared to the control without treatment (Figure 3A). Similar results were obtained after 30 min of treatment with N-CF, a potent HIF-2α antagonist, decreasing adhesion capacity of GSCs, thereby suggesting that the effects of ADA on cell adhesion could be through HIF-2α (Figure 3A, right bar). This suggests that adenosine depletion at 30 min was enough to decrease cell adhesion under hypoxia, which is an essential step in cell migration and motility. To confirm these results, a 3D-transwell migration assay was performed with U87-GSCs and PC-GSCs. As expected, hypoxia enhanced the migratory capacity of GSCs (Figure 3B), but surprisingly, adenosine depletion with ADA prevented the effect of hypoxia, both in U87-GSCs (Figure 3B) and PC-GSCs (Figure 3C). To evaluate the role of epithelial mesenchymal transition factors (EMT) in this process, verifying that under hypoxic conditions, ADA is able to decrease protein and mRNA levels of Snail and Twist1 in U87-GSCs and PC-GSCs (Figure 3D). Additionally, levels of EMT markers were measured by western blot. As expected, Zeb1 increased under hypoxia, which was not observed with ADA treatment (Figure 3E). As expected, ADA treatment under hypoxia promotes E-cadherin re-expression (Figure 3E), highlighting the role of this enzyme in prevents EMT. All together, these results confirm that the effects of hypoxia in cell adhesion/migration and EMT markers expression are adenosine/HIF-2α-dependent and can be prevented with ADA.

### 3.4. Adenosine Depletion Decreases GSCs Hypoxia-Dependent Cell Invasion

**Figure 4:**
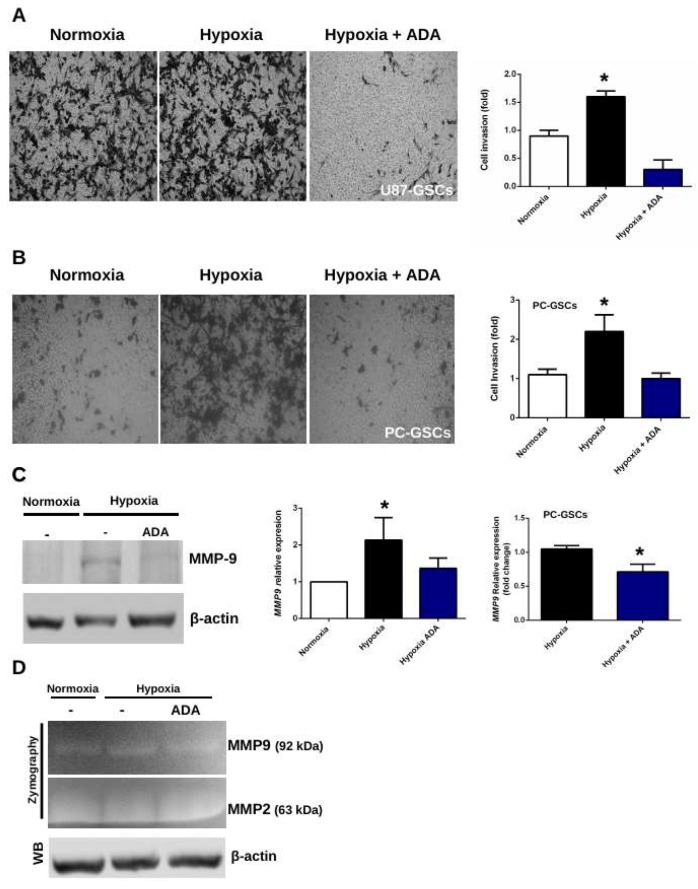
Effects of adenosine depletion on GSCs invasiveness. (**A**) Matrigel-coated transwell invasion assay of U87-GCSs in absence or presence of 1 U/mL ADA for 12 h. Cell invasion was plotted as fold change of the normoxic condition. (**B**) Same as in **a** but using PC-GSCs. (**C**) MMP-9 protein levels of U87-GSCs were analyzed by western blot and transcript levels of U87-GSCs and PC-GSCs were analyzed by RT-qPCR in the same treatmentconditions of **A**. **(D)** MMPs gelatinase activity was measured by zymography assay with GSCs extracellular medium. *n* = 3 * *p* < 0.05.

In addition to migration, cell invasion is a fundamental step for the infiltrationof GSCs into healthy brain tissue. To evaluate this, cells were seeded in matrigel-coated transwell chambers. As expected, hypoxia enhances cell invasion, probably due to the stabilization of HIF-2α. In contrast, ADA treatment prevents the effects produced by the low oxygen concentrations in both, U87-GSCs (Figure 4A) and PC-GSCs (Figure 4B), demonstrating that it is not just a cell line-dependent effect. These results are correlated with MMP-9 protein levels, which increase under hypoxia, but are reverted when extracellular adenosine is depleted (Figure 4C). In U87-GSCs and PC-GSCs, MMP9 transcript levels under treatment with ADA were also down-regulated (Figure 4C). To confirm the ability of GSCs to degrade extracellular matrix, a gelatinase assay was performed. As expected, MMP-9 gelatinase activity increased under hypoxia, which was prevented in treatment with ADA (Figure 4D). No changes were observed in MMP-2 activity, highlighting the relevance of MMP-9 in the infiltrative capacity of GSCs. All together, these results suggest that ADA decreases hypoxia/HIF-2α-dependent invasive phenotype mediated by MMP-9.

### 3.5. Adenosine Depletion Decreases GSCs In Vitro Tumorigenicity and Vincristine Chemoresistance

**Figure 5:**
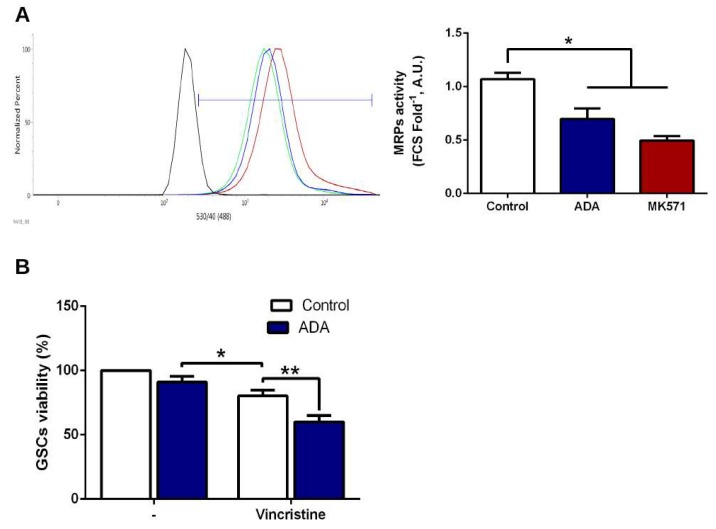
Effects of adenosine depletion on MRPs-mediated chemoresistance. (**A**) MRPs activity was analyzed by flow cytometry indirectly by CFDA accumulation of U87-GSCs in absence or presence of 1 U/mL ADA. 50 μM MK571 was used as control of MRPs inhibition. Histogram represents fluorescence of CFDA accumulation (left) and MRPs indirect activity was plotted as fold change of fluorescence^−1^(right). (**B**) GSCs viability was measured by MTS in absence or presence of 1 U/mL ADA and 100 nM Vincristine. *n* = 3, * *p* < 0.05; ** *p* < 0.01.

**Figure 6:**
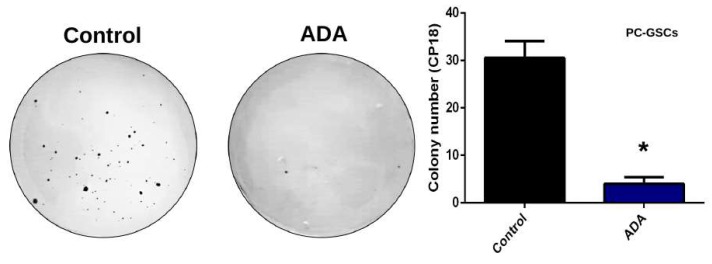
Effects of adenosine depletion on colony formation. U87-GSCs or PC-GSCs were seeded on soft agar plates in absence or presence of 1 U/mL ADA for 21 days. Colonies were counted and plotted. *n* = 3, * *p* < 0.05.

MRPs-mediated chemoresistance assays were performed under normoxia. ADA decreased MRPs extrusion capacity of a fluorescent substrate (CDFA), which is accumulated inside the cells by MRPs downregulation or by inhibiting its activity with MK571 (Figure 5A). ADA treatment is also capable of chemosensitizing GSCs to Vincristine treatment, a MRP known substrate, since the drug alone is capable of decreasing GSCs viability only up to 20%, while treatment with ADA enhances its effect on chemosensitizing GSCs to Vincristine (Figure 5B). Furthermore, we demonstrate that the addition of ADA is capable of decreasing colony formation in soft agar assays in PC-GSCs (Figure 6), demonstrating an effective method to decrease in vitro tumorigenicity of these cells responsible for tumor recurrence.

## 4. Discussion

It has long been known that adenosine plays an important role in the malignancy of GBM cells, specifically on stemness maintenance under hypoxic microenvironment [10]. Our group had previously reported that adenosine levels are elevated in GSCs and are further increased under hypoxic conditions [41]. Recently we reported that adenosine-dependent cell invasion under hypoxia is mediated primarily through its production by prostatic acid phosphatase (PAP), thereby enhancing signaling though its low affinity receptor A_3_AR [11]. However, until now, no therapy has been proposed to decrease cell invasion of hypoxic GSCs with a drug currently used in a disease or tested in clinical phases. Our group showed that A_3_AR blockade by MRS1220 antagonist, dramatically decreases cell invasion under hypoxic conditions [11] and can even promote chemosensitization to vincristine [9]. However, MRS1220 nor other commercial ARs antagonists are in clinical use, so transferring this research to current GBM treatments would take a long time and several clinical phases. Here, we targeted GSCs aggressiveness with recombinant adenosine deaminase (ADA), which is currently used in other diseases, such as severe combined immunodeficiency (SCID) [16]. The fact that ADA is a drug already tested may facilitate its transfer to clinical phases. It is important to highlight that extracellular adenosine levels of both normoxic (perivascular GSCs) and hypoxic GSCs are aberrantly elevated in relation to its differentiated counterpart (non-GSCs), so ADA treatment could be targeted both cell niches. However, this must to be tested by in vivo assays, evaluating the effect of ADA under a heterogeneous context as the tumor microenvironment. Here we show that the addition of 1 U/mL ADA was enough to decrease adenosine levels up to 75% in GSCs cultures under hypoxic conditions (Figure 1), demonstrating that with this method it is possible to decrease extracellular levels of this nucleoside even in conditions where it is aberrantly increased, such as hypoxic GSCs.

### 4.1. Adenosine Deaminase and Hypoxia-Dependent HIF-2α Stability

It has been established that the role of adenosine in cell invasion under hypoxic conditions would be mediated primarily by HIF-2α, which is expected because HIF-1α is more related to metabolic pathways regulation being stabilized at early stages of hypoxia, while HIF-2α is more related to chronic hypoxia and cell motility and invasion [34,35]. However, it is known that HIF-2α regulates the adenosine pathway through PAP expression [26], which is an ectonucleotidase sharing the same function of CD73 in the production of this nucleoside [11,20,42]. Here we show that adenosine depletion with ADA decreases HIF-2α protein levels, while no changes in transcript expression were observed (Figure 1), suggesting post-trascriptional adenosine-dependent regulation. It has been proposed that HIF-2α protein levels regulation is mainly dependent on its stability and not necessarily on its transcriptional up-regulation under hypoxia; furthermore, it has been reported that even in endothelial cells under hypoxia transcript levels decrease while protein amount increases [43]. In MG-132 assays, ADA was unable to reverse HIF-2α accumulation under proteasome inhibition, suggesting that in GSCs adenosine would promote accumulation of this factor through inhibition of its protein degradation rather than transcriptional regulation. Here, we suggest a positive regulatory loop for HIF-2α stabilization under hypoxia and adenosine production/signaling. To determine adenosine-dependent stability was not possible due to HIF-2α fast degradation rate, which already decreases dramatically at 5 min under treatment with cycloheximide (that inhibits protein production) (Figure 2).

### 4.2. Adenosine Deaminase in GSCs Invasive Phenotype Under Hypoxia

Although higher effects on the decrease of extracellular adenosine levels were observed at 24 h, only 30 min of treatment of hypoxic GSCs with ADA were enough to decrease adhesion to 50% (Figure 3) and only 6 h of treatment already reduced 3D-cell migration in transwell assays, even in GSCs derived from primary cultures. Similar results were obtained in matrigel-coated invasion chambers with 12 h of ADA treatment. These results suggest that A_3_AR signaling is downregulated, however a role of the A_2B_AR is not ruled out, which is also a low affinity adenosine receptor and its signaling is enhanced under hypoxic microenvironment, being also regulated by HIF-2α [26]. A_2B_AR is expressed and activated in several cancer models, including GBM [23,44]. HIFs stabilization under hypoxia increases A_2B_AR expression, thereby enhancing adenosine signaling under hypoxic microenvironments [27,45]. A_2B_AR activation increases the infiltration of GBM cells into the healthy brain tissue and its blockage decreases MMPs expression and activity [46]. This evidence and our results suggest a role of A_2B_AR and A_3_AR in enhanced infiltrative phenotype of GBM and GSCs. We suggest that adenosine depletion by ADA downregulates A_2B_AR/A_3_AR signaling, in order to decrease migratory and invasive capacity of GSCs under hypoxic conditions. RNAseq analysis confirmed that under hypoxia and blocking A_3_AR, *Twist1* and *Snail* transcript levels decreased, which was confirmed by western blot [11], demonstrating a relationship between adenosine signaling and EMT marker expression. Here, these results were further confirmed in hypoxic GSCs treated with ADA, confirming that the effects of adenosine depletion on hypoxia may modify the expression of Twist1, Zeb1 and Snail EMT markers, which could help to explain the decrease in adhesion and cell migration described previously.

### 4.3. Adenosine Deaminase in GSCs Chemoresistance and Clonogenicity

Previously, our group confirmed that A_3_AR blockage decreased the expression of the ABC transporter MRP1, which is highly expressed in GBM, promoting chemoresistance to Vincristine [9,47]. Here we show that ADA is also able to decrease MRPs activity, conferring chemosensitivity to vincristine (Figure 5). These effects shown here could be attributable to MRP1, which is regulated by adenosine signaling [9], but it has been suggested that its effects are compensated by MRP3, another ABC transporter sharing target drugs with MRP1 [48,49]. This, together with the decrease in colony formation capacity, suggest that ADA treatment would not only decrease cell invasion, but also tumor aggressiveness bringing about a new therapeutic strategy for this deadly disease. Now, the challenge will be to expand this research with in vivo studies to evaluate GSCs infiltration and develop ADA delivery methods.
